# Viscoelastic Numerical Simulation Study on the Co-Extrusion Process of Tri-Composite Tire Tread

**DOI:** 10.3390/ma16093301

**Published:** 2023-04-22

**Authors:** Guo-Lin Wang, Hua-Jian Zhou, Hai-Chao Zhou, Chen Liang

**Affiliations:** School of Automotive and Traffic Engineering, Jiangsu University, Zhenjiang 212013, China

**Keywords:** die swell, co-extrusion process, PTT constitutive model, process parameters, die structure, numerical simulation

## Abstract

The co-extrusion process is widely used to produce composite tire treads with better performance. This study investigated the rubber co-extrusion flow process and quality influencing factors of tri-composite tire tread through numerical simulation and experimental methods. Here, RPA 2000 rubber processing analyzer was used to carry out rheological tests on the three rubber materials, the PTT viscoelastic constitutive model was fitted, and the fitting curves were in good agreement with the test data. Then, a three-dimensional viscoelastic numerical simulation model of the tri-composite tread co-extrusion process was established using Ansys Polyflow software. The parameter evolution technique is adopted in the model establishment to improve the calculation convergence. In addition, a global remeshing function is used to avoid excessive mesh deformation. A co-extrusion experiment is conducted to verify the model’s accuracy using a tri-screw extruder. The extruded tread size error rate between the experiment and simulation is less than 6%. The variation of the velocity field, pressure field and shear rate field during extrusion is analyzed, and the formation mechanism of die swell is explained simultaneously. Finally, the influence of process parameters (inflow rate and traction speed) and die structure (convergence angle and thickness) on the extruded tire tread shape and quality was investigated, which can provide theoretical guidance for improving tread quality and production efficiency. Furthermore, the numerical simulation method can assist the design of the die plate in enhancing the efficiency of the die plate design.

## 1. Introduction

With the rapid development of the automobile industry in recent years, people have put forward higher requirements for the performance of tires. Tire manufacturing companies use co-extrusion molding technology [1] to produce composite tire treads with better performance. Co-extrusion process refers to using multiple screw extruders to compound different types of rubber materials at the die plate of the extruder head to form a composite tread [2]. However, as a kind of polymer material, extrusion rubber exhibits rheological properties such as shear thinning and die to swell [3]. At the same time, the invisibility of the flow process of rubber also increases the difficulty of die structure design and process parameters formulation. The traditional “trial and error” die design method requires multiple extrusion tests and die modification [4]. This design approach has significant drawbacks, such as a long design cycle and poor applicability. Foremost, it lacks a theoretical basis. Overall, it leads to low production efficiency and poor-quality stability of the extruded tread.

With the development of computer technology, the numerical simulation method can be used to simulate the co-extrusion process, which can predict the profile of the extruded tread and obtain the distribution and change of the velocity, pressure, and shear rate in the process of rubber flow, thus providing a theoretical basis for the design and optimization of the die structural and process parameters. In the numerical simulation study of the polymer co-extrusion process, Takase et al. [5] simulated the flow of two viscoelastic fluids in a square passage based on the finite element method. They found that the difference in viscosity and elasticity between the two materials would lead to the deformation of the material interface. With the development of flow, one material would gradually wrap the other. Anderson et al. [6] simulated the co-extrusion flow in square and rectangular channels. They determined the location of the material interface by mapping method, finding that the secondary flow of polymer melt has an important influence on the shape of the interface. Sharma et al. [7] used the Carreau-Yasuda constitutive model to simulate the variation of velocity, pressure and die swelling in the co-extrusion process of special-shaped rubber strips, providing theoretical methods for the design of die opening shape and the improvement of extrusion quality. Liang [8] studied the influence of die Angle on the extrusion expansion rate. It found that the convergent flow at the die entrance and the elastic recovery of shear and tensile deformation caused by shear strain in the die flow after melt leaves the die should be the main factors causing die swelling. Sunwoo et al. [9] used PTT (Phan-Thien–Tanner) viscoelastic constitutive model to simulate the flow of two kinds of adhesives in the rectangular die. They studied the influence of viscoelastic properties of adhesives, such as shear rate and second normal stress difference, on the interface curvature of extrudates. Liu et al. [10] established a viscoelastic numerical simulation model of double composite extrusion by using the Navier slip model, focusing on the influence of the wall slip coefficient on the deflection degree of the extruder interface. The results show that the deflection degree of the interface increases with the increase of the slip coefficient.

Previous simulation studies mainly focused on single and double extrusion processes considering a relatively simple die structure. However, the co-extrusion process of tri-composite tire tread is more and more widely used in actual production. Compared with double compound extrusion, the flow of rubber melt in the triple compound extrusion process is more complex, so it is more complex to establish and calculate the numerical simulation model. In this paper, the rheological properties of rubber were tested by RPA 2000 rubber processing analyzer [11]. Then a three-dimensional viscoelastic numerical simulation model of the co-extrusion process of a tri-composite tread rubber compound from the extruder head was established by using PTT viscoelastic constitutive model, and the accuracy of the model was verified by co-extrusion experiment. The influences of two process parameters (inflow rate, traction speed) and two die structure parameters (die thickness and die convergence angle) on extrusion tread profile and extrusion tread quality were investigated, which can be used to guide the design of die structure and process parameters in actual production. It has practical significance in improving product quality and efficiency.

## 2. Numerical Simulation Model

### 2.1. Mathematical Model

**Figure 1:**
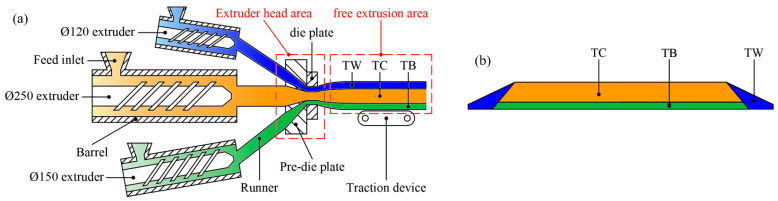
Extruder structure and extruded tread diagram (**a**) extruder structure; (**b**) extruded tread.

Figure 1a shows the structure of the tri-screw extruder, which is composed of three screw extruders of different specifications, runners, pre-die plate, die plate and traction devices. Notice that the actual structure of the extruder is more complex than indicated. The co-extrusion process’s essential features (stages) are summarized as follows. First, three rubber materials enter the extruder as solid sheets from their respective feed inlets at room temperature. Then, they suffer the shear and stretch action formed by screw rotation in the barrel. Next, due to the viscous heating effect of the rubber material and the heating/cooling effect of the extruder, the temperature of the rubber material will gradually rise and stabilize at 80 °C to become a molten plastic state. Then it flows through the runner and the pre-die plate and converges in the die plate to form the tri-composite tire tread. There, the temperature of the rubber material is about 110 °C. Finally, it is picked up by the traction device for cooling and other subsequent processes. Figure 1b is the section diagram of the extruded tread. TC, TB, and TW represent crown, wing, and base rubber, respectively. The crown rubber is the direct contact part between the tire tread and the ground, the wing rubber is distributed on both sides of the tread, and the base rubber is located under the crown rubber.

The pre-die plate and the die plate constitute the extruder head. Based on the flow characteristics of the rubber melt in the extruder head and the free extrusion area [12], the following assumptions are made: 1. The flow process is isothermal steady laminar flow; 2. The inlet flow is fully developed, ignoring the inertia force and volume force; 3. The melt is incompressible and insoluble [13]. Based on the above assumptions and simplification, the governing equation of the flow field is shown in Equations (1) and (2).

1. continuity equation
  (1) $$\nabla \cdot v_{k}=0\;\;k=I,\mathrm{II},\mathrm{III}$$
2. momentum equation
  (2) $$\rho_{k}(v_{k}\cdot \nabla )v_{k}=-\nabla p_{k}+\nabla \cdot \tau_{k}\;\;k=I,\mathrm{II},\mathrm{III}$$

Here ∇ is the Hamiltonian; *v* is the velocity vector; *ρ* is the rubber density; *p* is the hydrostatic pressure and isotropic; *τ* is the deviatoric stress tensor; *k* represents three types of rubber, respectively.

### 2.2. Constitutive Model and Parameters

To simulate the die swell process of the rubber melt after extrusion from the die, the Phan-Thien-Tanner (PTT) [14] differential viscoelastic constitutive model is selected. PTT constitutive model can characterize the shear thinning and die swell characteristics of polymer fluid [15]. The expression is shown in Equation (3).

(3) $$\begin{array}{l} \tau =\tau_{1}+\tau_{2}\\ \exp\left[\frac{\varepsilon \lambda }{\eta_{1}}tr(\tau_{1})\right]\tau_{1}+\lambda \left[\left(1-\frac{\xi }{2}\right)\overset{\nabla }{\tau_{1}}+\frac{\xi }{2}\overset{\Delta }{\tau_{1}}\right]=2\eta_{1}D\\ \tau_{2}=2\eta_{2}D \end{array}$$

Here, τ is the stress tensor; $\tau_{1}$ and $\tau_{2}$ are the elastic and viscous components of the stress tensor, respectively; *ε* is the material parameter related to stretching property; *ξ* is the material parameter related to shear property; *λ* is the relaxation time; $\eta_{1}$ and $\eta_{2}$ are the elastic and viscous components of the zero-shear viscosity respectively; *D* is the rate of deformation tensor. ∇ and Δ are the lower-convected and upper-convected time derivation, respectively.

**Figure 2:**
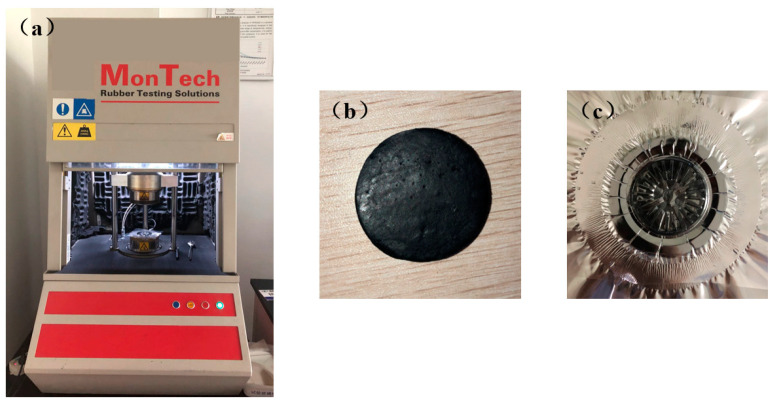
RPA 2000 rubber processing analyzer and rubber sample (**a**) RPA 2000 (**b**) rubber sample before the test (**c**) rubber sample after the test.

As shown in Figure 2, to obtain the experimental rheological data of the rubber and fit the PTT constitutive model, the RPA 2000 processing analyzer was used to perform frequency scanning tests in accordance with ISO standards [16]. The frequency scanning range was 0.03–33 Hz, and the oscillation strain rate was 12%. Since the rubber temperature at the extruder head is about 110 °C, the test temperature was set to 110 °C. Three tests were carried out for each rubber to reduce the error.

**Figure 3:**
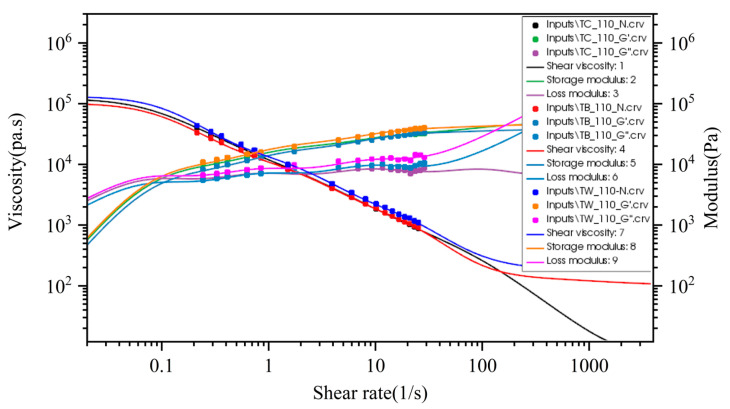
PTT fitted curves and experimental data.

The polymat [17] module under the fluid simulation software Ansys is used to fit the PTT constitutive model. The PTT fitted curves (lines) and the experimental data (solid dots) are depicted in Figure 3. It can be seen that the fitting curves are in good agreement with the test data. PTT constitutive model parameters are listed in Table 1.

### 2.3. Geometry Model and Mesh Division

**Figure 4:**
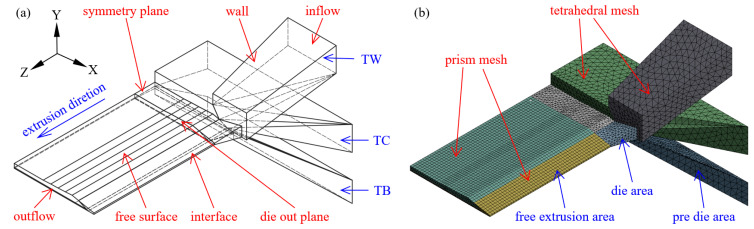
Three compound extrusion physical models (**a**) geometry model; (**b**) mesh model.

Taking the extruder head and free extrusion areas as the modeling objects due to the geometric symmetry, to improve the calculation efficiency, a 1/2 model is established, as shown in Figure 4a. To ensure that the profile of the extruded tread is stable, the length of the free expansion area is 150 mm. The grid model is shown in Figure 4b. Due to the irregular structure of the pre-die and the die, it is discretized into a tetrahedral non-structural grid. Due to the deformation problems of the free surface and interface in the free extrusion area, the remeshing technique [18] is used to improve the convergence. Therefore, the discretization is made into the pentahedral prismatic grid, and all areas were set with second-order node grids to improve calculation accuracy.

### 2.4. Flow Boundary Conditions

Flow boundary conditions were set according to Figure 4a, and the process parameters are shown in Table 2. *f_n_*, *f_s_*, *v_n,_* and vs. represent the normal force, tangential force, normal velocity, and tangential velocity, respectively.

1. Inlet: the total inlet volume flow rate is Q, and the flow is fully developed.
2. Wall: Generalized Navier’s law [19] was adopted to characterize the slip of the rubber melt at the wall, as shown in Equation (4) below:
  (4) $$F(v)=-kv^{e}$$
  Here, *F*(*v*) is the wall slip resistance; *k* is the slip coefficient, taking the value of $1\times 10^{6}$; *e* is a dimensionless number; *v* is the velocity of rubber melt at the wall surface.
3. Interface: Surface tension is neglected, and there is no slip at the interface. Dynamic conditions: $f_{s}^{I}=f_{s}^{\mathrm{II}}=f_{s}^{\mathrm{III}}$; Kinematic conditions: $v_{n}=0$.
4. Free surface: Dynamic conditions: $f_{n}=f_{s}=0$; Kinematic conditions: $v_{n}=0$.
5. Symmetry plane: Dynamic conditions: $f_{s}=0$; Kinematic conditions: $v_{n}=0$.
6. Outlet: Setting traction speed *v_z_*.

### 2.5. Calculation Strategy

The polymer simulation software Ansys Polyflow establishes a numerical simulation model. Due to the use of the PTT viscoelastic constitutive model and relatively complex geometric structure, the numerical calculation may be difficult to converge [20], so it is necessary to set up the appropriate numerical algorithm. To improve convergence, a progressive calculation model is established, and a progressive algorithm is set for the relaxation time parameter of the PTT model. The interpolation scheme selects DEVSS SU [21] and mini-element to solve the stress, velocity and pressure field. In addition, the mesh deformation of each interface and free surface in the free extrusion area of the wall may cause the mesh to be too large, so the Optimesh-3D remeshing technology [22] is used to continuously reset and optimize the mesh.

## 3. Results and Discussion

### 3.1. Model Verification

**Figure 5:**
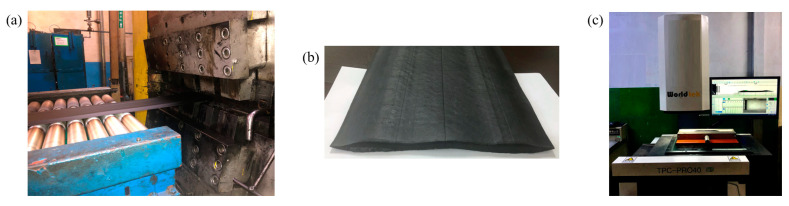
Co-extrusion experiment (**a**) Extruder; (**b**) Extruded tread; (**c**) Section analyzer.

To verify the validity of the numerical simulation model, a tri-screw extruder [23] was used to carry out the co-extrusion experiment under actual production conditions. Figure 5 illustrates the experimental process. First, when the shape of the extruded tread is stable, tools are used to cut a piece of tread from the production line quickly. The cut tread is shown in Figure 5b. Then a section analyzer is used to scan the profile of the tread section and record the dimensional data at key positions.

**Figure 6:**
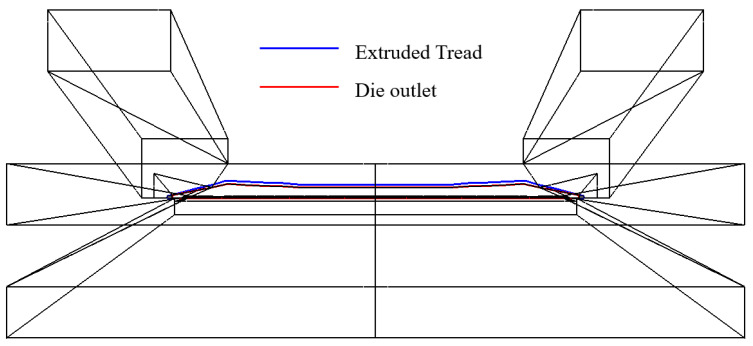
Simulated extruded tread profile.

Figure 6 shows the profile of extruded tread obtained by simulation. The navy blue and red lines are the die outlet and extruded tread profiles, respectively. It can be observed that the profile of the extruded tread extends outward significantly compared with the profile of the die outlet. The phenomenon is called die swelling.

**Figure 7:**
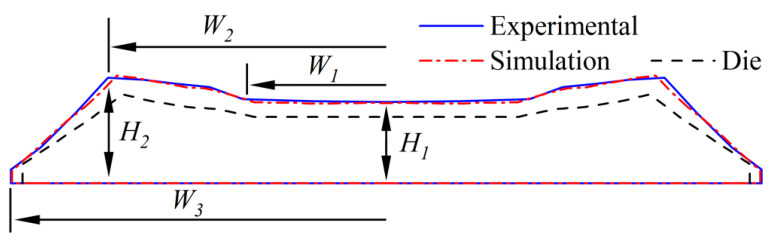
Comparison of simulated and experimental tread profile.

The comparison between the simulation and the experimental extruded tread profile is shown in Figure 7. Table 3 shows the size deviation rate of the key positions of the tread and die swell rate deviation. It can be seen from Figure 7 and Table 3 that the simulation results are in good agreement with the experimental results, and the errors are both lower than 6%, so the model is effective. The reasons for the error include (1) The size error when using laser scanning equipment to map the profile of the die; (2) the rheological test and constitutive model fitting error; (3) The influence of temperature variation on rubber flow which is not considered in the simulation.

The die swell rate [24] can be calculated by Equation (5):

(5) $$B=\frac{\left(S-S_{0}\right)}{S_{0}}\times 100\%$$

Here, *B* is the die swell rate; *S* is the sectional area of extruded tread; *S*_0_ is the area of the die outlet surface.

### 3.2. Die Swell Analysis

**Figure 8:**
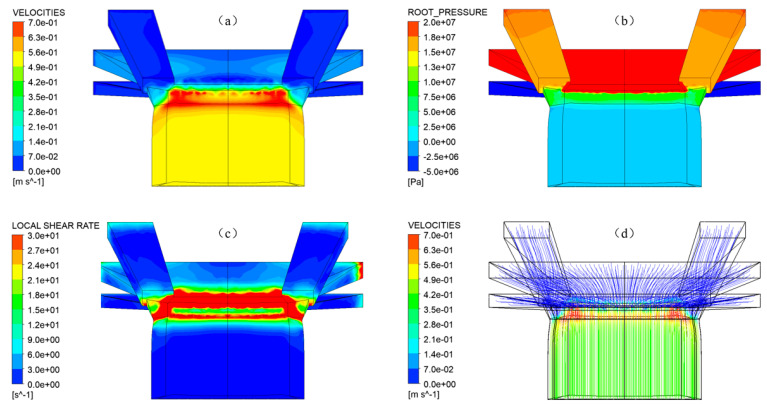
Countor results (**a**) velocities; (**b**) pressure; (**c**) shear rate; (**d**) streamline velocity.

Figure 8 shows the results obtained from the co-extrusion simulation. The red and blue zones represent the highest and lowest velocity, pressure and shear rate, respectively. Figure 8a represents that the velocity is higher in the die and gradually decreases after leaving the die. Figure 8b shows that the pre-die internal pressure of the crown rubber is the largest because the crown rubber flow rate is significantly higher than the other two rubber compounds. When the rubber is extruded from the die, the pressure drops rapidly to zero. Figure 8c clearly shows that the shear rate in the die’s entrance and exit wall areas is the highest, which indicates that the flow fluctuation of rubber at the entrance and exit areas of the die is very violent.

**Figure 9:**
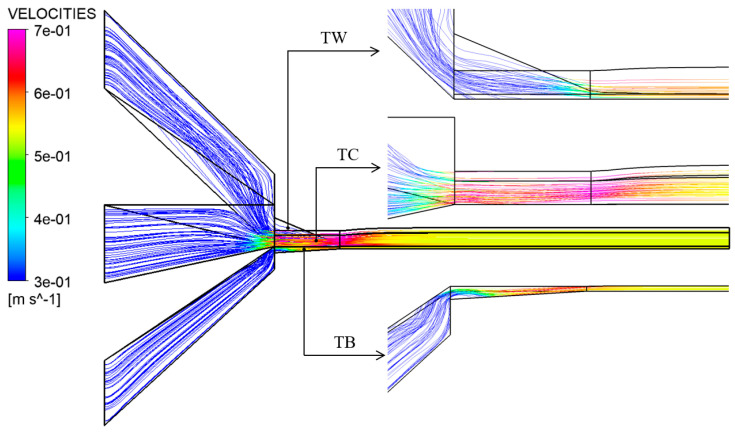
Simulation results of velocity streamlines.

**Figure 10:**
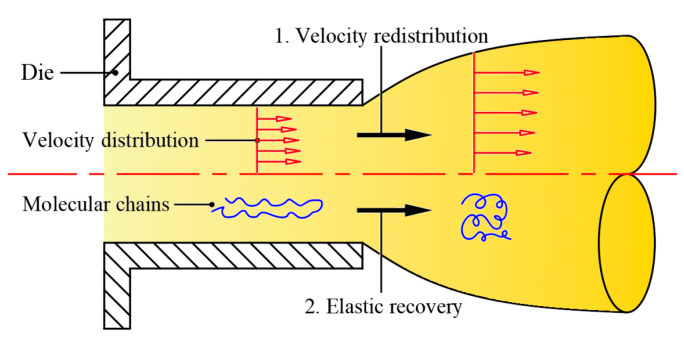
Forming mechanism of die swell.

Figure 9 shows the left view of the velocity streamlines obtained by numerical simulation, from which we can observe the die swell phenomenon of the rubber melt after it is extruded from the die. As shown in Figure 10, the formation mechanism of die swell can be explained as the following two points: one is that after the rubber is extruded from the die, the velocity is redistributed due to the loss of wall shear and constraint; the other is that the rubber melt will form convergent flow after entering the die from the pre-die, which can be observed from Figure 9. At the same time, the originally entangled rubber macromolecular chains are elongated by shearing and stretching, thus accumulating the normal stress [25]. When the rubber is extruded from the die, the normal stress will gradually release. The macromolecular chain will return to the original entangled state, forming the die-swell phenomenon on the macro level.

**Figure 11:**
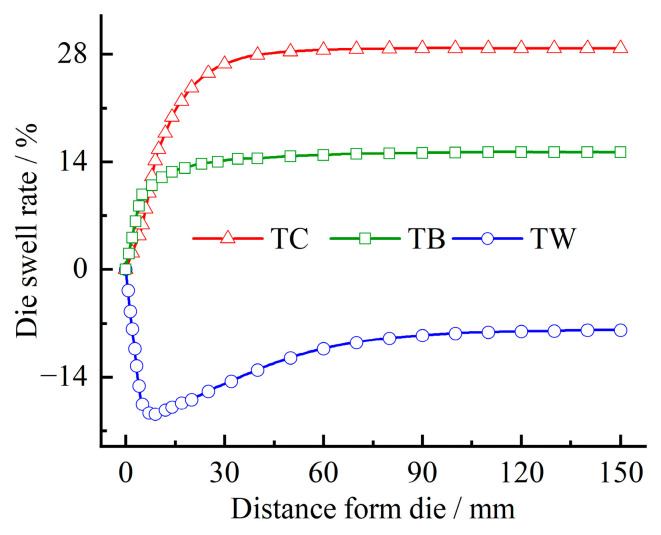
Variation of die swell rate with distance from the die.

Figure 11 shows the variation of the die swell rate of the three rubber materials with the distance from the die. It can be seen from the figure that the die swell rate of the crown rubber TC and the base rubber TB increases rapidly after extruding from the die. The increasing trend gradually slowed, and the die swell rate reached stability at nearly 80 mm. On the other hand, the die-swell rate of the wing rubber TB varies significantly. The die swell rate decreases rapidly within 10 mm after leaving the die, then increases slowly and reaches a stable die swell rate of nearly 100 mm. The special variation process of wing rubber may be caused by the swelling of the crown rubber after release from the die has a squeezing effect on the wing rubber. The finally stabilized die swell rates of the three rubber materials are quite different, which are 28.7%, −7.8% and 15.3%, respectively. This is because the rheological properties of the three rubber compounds and the structure of the pre-die and die through which they flow are different, resulting in different flow characteristics of the compounds, leading to different die swell profiles.

### 3.3. Co-Extrusion Influencing Factors

#### 3.3.1. Process Parameters

The main process parameters that can be adjusted in co-extrusion production are inflow rate and traction speed. The inflow rate is controlled by adjusting the screw rotating speed, and the traction speed is controlled by adjusting the motor speed of the traction device. To explore the influence of inflow rate and traction speed on the co-extrusion process, as shown in Table 4, five plans were set up, among which plan 3 is the original plan. Plans 1, 2 and 3 are to keep the traction speed unchanged and change the inflow rate; Plans 3, 4 and 5 are to keep the inflow rate unchanged and change the traction speed.

**Figure 12:**
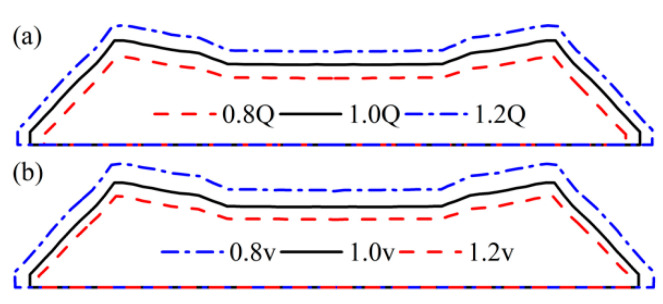
Extruded tread profile under different process parameters (**a**) inflow rate; (**b**) traction speed.

Figure 12 shows the profiles of the extruded treads under different process parameters. It can be seen from Figure 12a that with the increase of the inflow rate, the tread profile uniformly expands outward, indicating that the die swell rate increases with the rise in the inflow rate; In Figure 12b, the tread profile shrinks inward with the increase of the traction speed, this indicates that increasing the traction speed will reduce the die swell rate.

**Figure 13:**
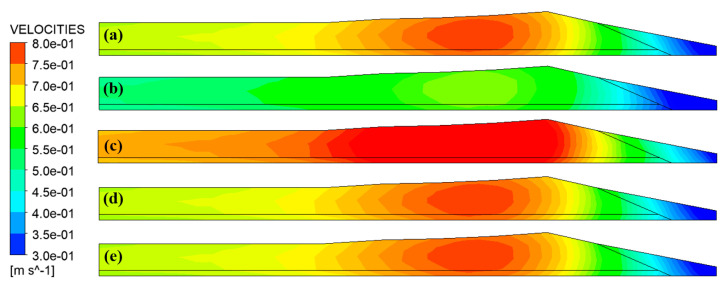
Velocity contour of die outlet (**a**) 1.0 Q, 1.0 v; (**b**) 0.8 Q, 1.0 v; (**c**) 1.2 Q, 1.0 v; (**d**) 1.0 Q, 0.8 v; (**e**) 1.0 Q, 1.2 v.

Figure 13 is the velocity contour of the die outlet. It can be observed that the velocity distribution on the outlet of the die shows a trend of gradually increasing from the left and right sides to the inside, and the maximum velocity appears in the area near the left side of the tire shoulder. In addition, the velocity of the wing tip area is significantly lower than that of other areas, indicating that the fluidity of the rubber in this area is very poor. This is due to the excessive flow resistance caused by its relatively narrow flow channel. The wing tip is where extruded tread quality problems such as shark skin and melt fracture [25] often occur. Comparing Figure 13a–c, it can be analyzed that the inflow rate has a more obvious influence on the velocity distribution on the die outlet. As the inflow rate increases, the velocity also increases; However, by comparing Figure 13a,d,e, it can be noticed that the traction speed has little effect on the velocity distribution because the traction speed is controlled by the traction device, which is located at a certain distance from the die. Hence, the influence on rubber flow in the die is limited.

In the process of co-extrusion, the uniformity of rubber flow will affect the uniformity of the material distribution of the extruded tread, and the uniformity of the tread material distribution is related to the performance of the finished tire [26]. Therefore, the flow uniformity of rubber compounds is taken as an evaluation index of extruded tread quality, and the variance of velocity is calculated by the Formula (6).

(6) $$\sigma_{V}{}^{2}=\frac{\sum {}_{i=0}^{N}{\left(v_{i}-\bar{v}\right)}^{2}}{N}$$

Here, $\sigma_{V}{}^{2}$ is the velocity variance, *N* is the number of points taken, $v_{i}$ is the velocity value of the *i*-th point, and $\bar{v}$ is the velocity mean.

**Figure 14:**
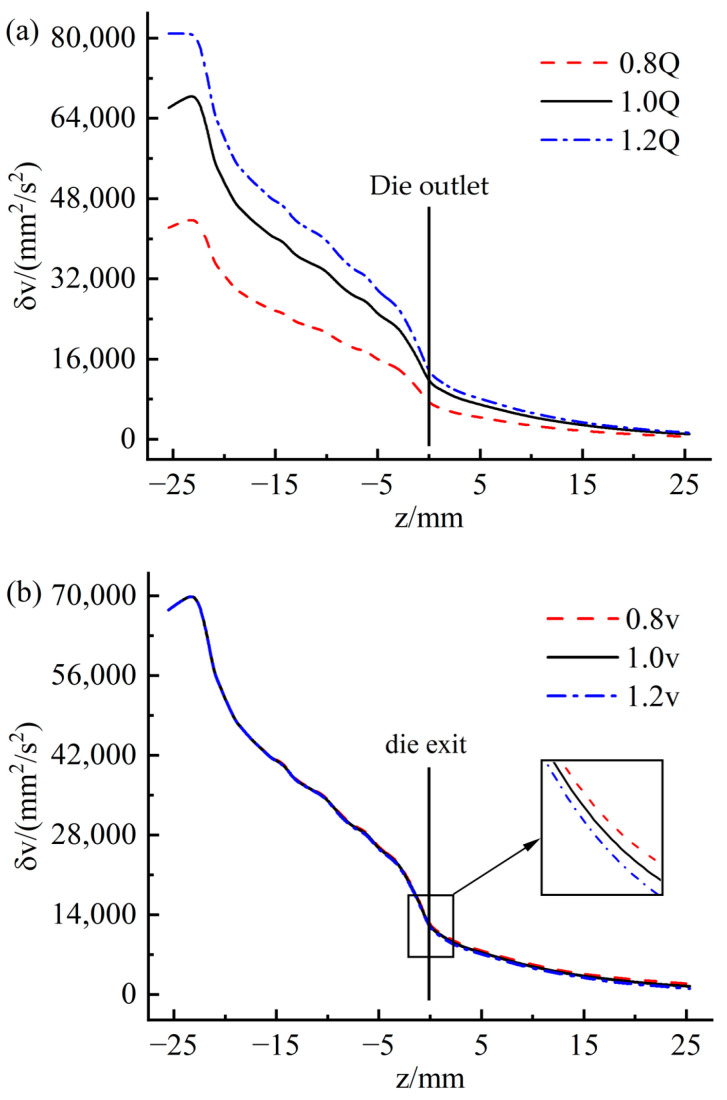
Variation of velocity variance along extrusion direction (**a**) flow rate; (**b**) traction speed.

Figure 14 shows the variation of velocity variance at different sections inside and outside the die along the extrusion direction. The sections of z = −25 and z = 0 represent the inlet and outlet of the die, respectively. It can be observed that the overall velocity variance along the extrusion direction presents a gradual downward trend. The decline is relatively rapid in the die, and the downward trend slows down after leaving the die. This indicates that the flow uniformity of the rubber melt increases gradually along the extrusion direction. The fluctuation of the velocity variance was observed in the 10 mm area from the inlet of the die, which is caused by the convergent flow of the rubber melt when it enters the die from the pre-die. Figure 14a represents that with the increase of the inflow rate, the flow uniformity of the rubber melt decreases. Unexpectedly, Figure 14b indicates that traction speed weakly influences the flow uniformity of the rubber to melt inside and outside the die.

During the flow process of the rubber melt, the shear rate is positively correlated with the shear stress [27]. If the shear rate at the die wall is too high, the rubber melt will break through the wall adhesion limit, and slip [28] will occur, resulting in surface quality problems of extruded tread, such as sharkskin [29] and melt fracture [30]. Therefore, the average shear rate at the wall is selected as another evaluation index for the quality of the extruded tread. The average shear rate is calculated by the Formula (7).

(7) $$\bar{\gamma }=\frac{\sum {}_{i=0}^{N}\gamma_{i}}{N}$$

where $\bar{\gamma }$ is the mean value of the shear rate, *N* is the number of points taken, and $\gamma_{i}$ is the shear rate value of the *i*-th point.

**Figure 15:**
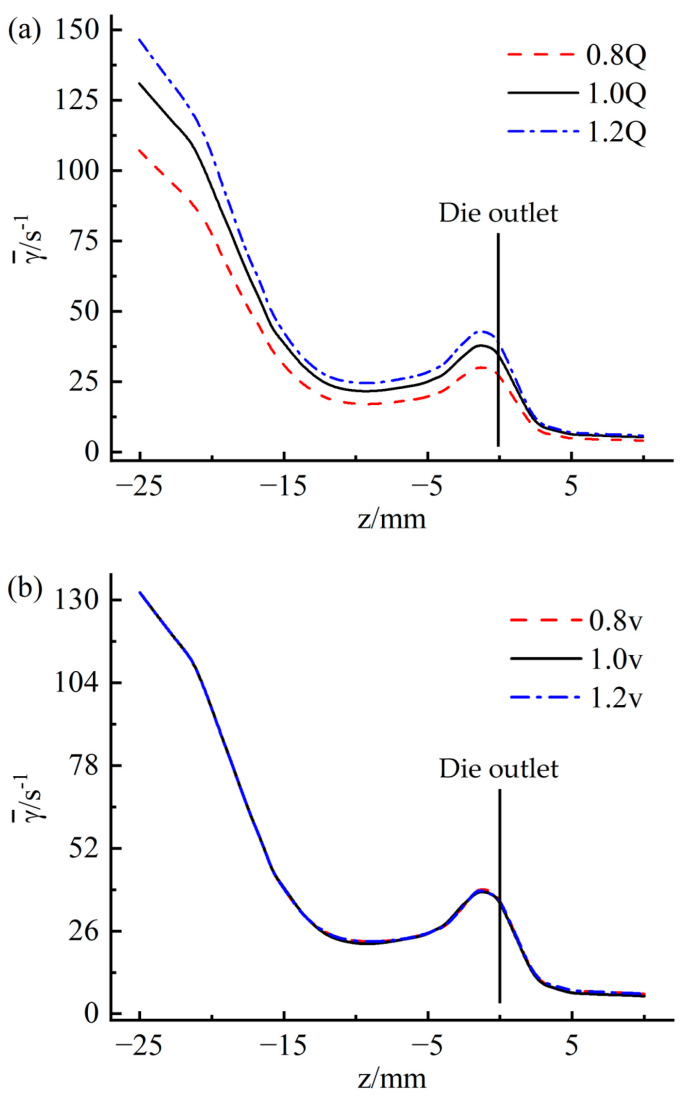
Variation of average shear rate along extrusion direction (**a**) flow rate; (**b**) traction speed.

Figure 15 shows the variation of the mean shear rate on the wall at different positions along the extrusion direction. On the whole, it can be seen from the figure that the wall shear rate shows a gradual downward trend along the extrusion direction. However, a sharp increase in shear rate was observed in the range of 5 mm from the die outlet, corresponding to that shown in Figure 8c. The sharp increase in shear rate may be caused by increased velocity caused by stress release when the rubber is extruded from the die. Figure 15a indicates that the wall shear rate increases as the inflow rate increases. Thus, the possibility of quality problems with the extruded tread will also increase. Observing Figure 15b, it can be found that the traction speed has very little effect on the shear rate, which is consistent with the law observed in Figure 14b.

In actual production, to ensure that the size of the extruded tread is constant, the inflow rate and the traction speed are adjusted synchronously. According to the influence of the above-mentioned inflow rate and traction speed on the quality of the extruded tread, it can be concluded that the excessively high inflow rate will lead to a decrease in the quality of the extruded tread, and a decrease in the inflow rate means a decrease in production efficiency. Therefore, it is necessary to reasonably formulate process parameters to obtain a balance between production quality and production efficiency.

#### 3.3.2. Die Structure

**Figure 16:**
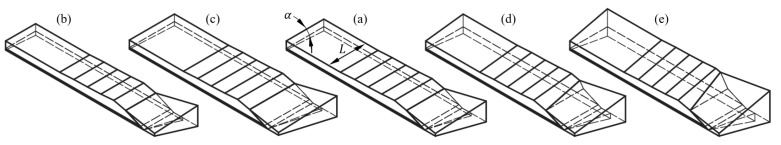
Die structure design (**a**) T = 25 mm, α = 3°; (**b**) T = 15 mm, α = 3°; (**c**) T = 35 mm, α = 3°; (**d**) T = 25 mm, α = 13°; (**e**) T = 25 mm, α = 23°.

To explore the influence of the die structure on the extrusion process, the die thickness and convergence angle were modified, respectively, as shown in Figure 16 and Table 5. Plan a is the initial die structure, the thickness T = 25 mm, and the convergence angle α = 3°; Plan b and c keep the convergence angle constant and change the thickness; Plan d and e keep the thickness constant and change the convergence angle.

**Figure 17:**
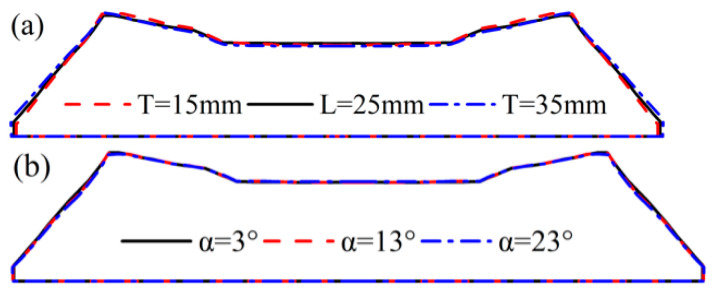
Extrusion profile under different die structures (**a**) die thickness; (**b**) die to converge angle.

Figure 17 is the extruded tread profile under different die structures. Figure 17a shows that the die thickness has little effect on the profile of the extruded tread. With the increase of die thickness, the wing rubber area slightly expands outward, and the crown rubber area shrinks inward. Figure 17b indicates that the convergence angle of the die has almost no effect on the profile of the extrusion section, and only the profile of the wing rubber TW area shrinks slightly inward with the increase of the die convergence angle. In contrast, the profile of other areas remains unchanged.

**Figure 18:**
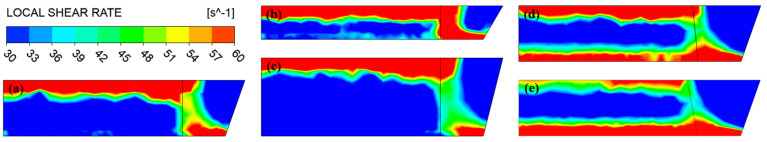
Shear rate contour of die upper wall (**a**) T = 25 mm, α = 3°; (**b**) T = 15 mm; (**c**) T = 35 mm; (**d**) α = 13°; (**e**): α = 23°.

Figure 18 is the contour of the upper wall of the die. It can be seen from Figure 18a–c that with the increase in die thickness, the shear rate at the die exit area decreases slightly. By comparing Figure 18a,d,e, it can be noticed that with the increase of the convergence angle of the die, the area of high shear rate shifts from the die’s entrance to the die’s exit. The exit of the wing rubber is always a high shear rate area, and the wing rubber is also the area where quality problems often occur in actual production.

**Figure 19:**
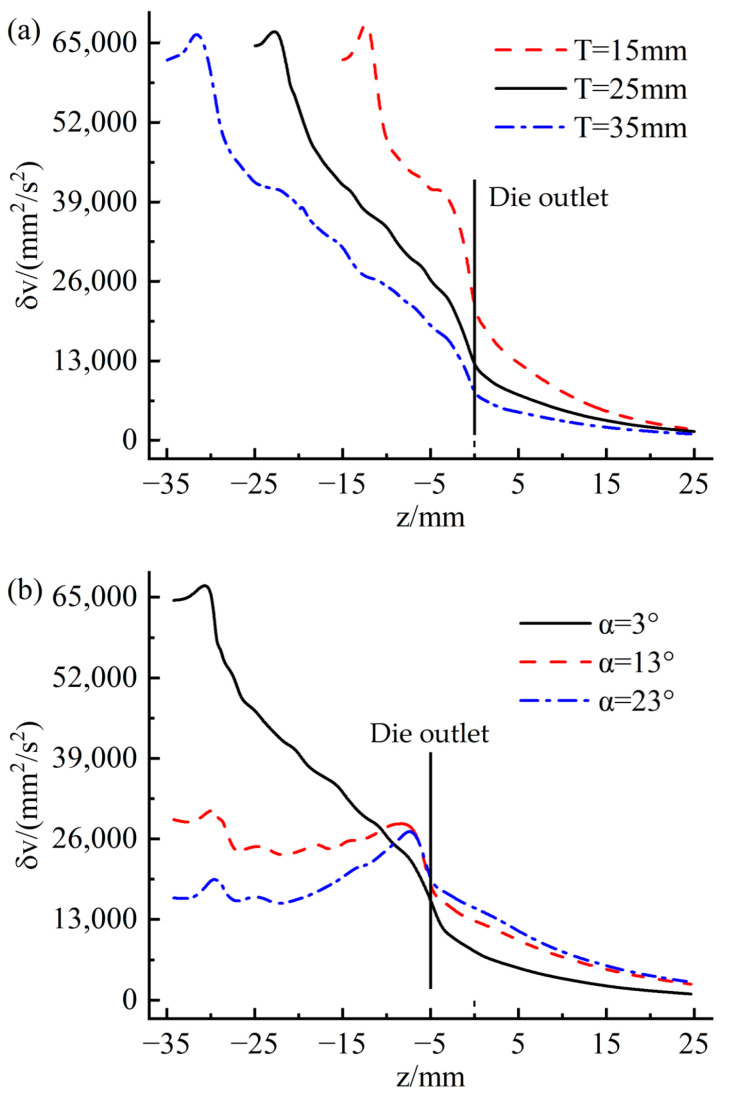
Variation of velocity variance along extrusion direction (**a**) die thickness; (**b**) die to converge angle.

The variation of velocity variance at different positions along the extrusion direction is presented in Figure 19. It is evident from Figure 19a that the increase in the thickness of the die leads to a decrease in the velocity variance, indicating that the flow uniformity has improved. This is because the increased thickness of the die gives the rubber melt more time to stabilize the flow. Figure 19b shows that the change in the convergence angle causes apparent fluctuations in the variance of the velocity inside and outside the die. With the increase of the convergence angle of the die, the variance of the flow velocity in the inlet area of the die decreases obviously, and the variance of the flow velocity in the outlet area of the die increases slightly. This is due to the different convergence angle designs resulting in a large difference in the flow characteristics of the rubber melt when it enters and leaves the die.

**Figure 20:**
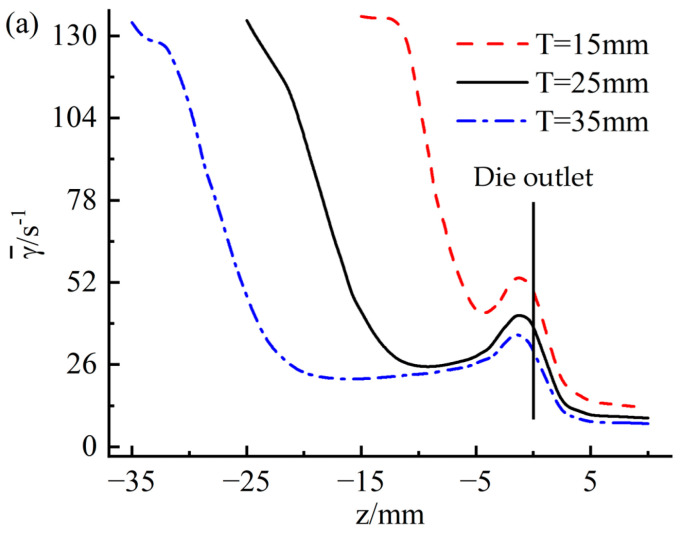

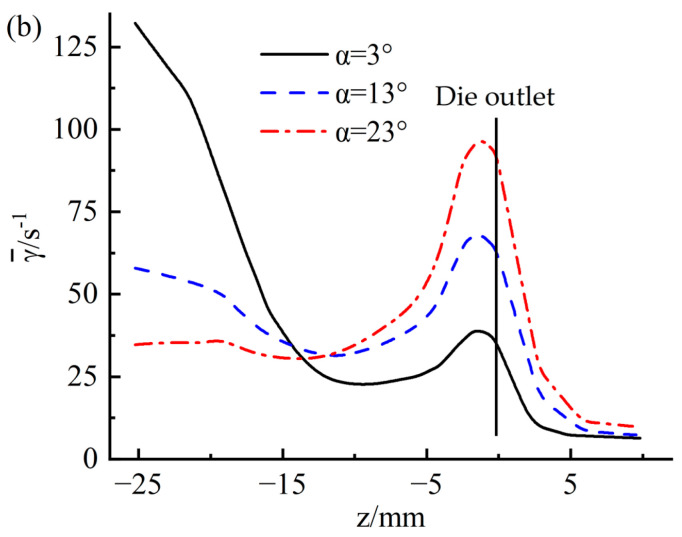
Variation of mean shear rate along extrusion direction (**a**) die thickness; (**b**) die to converge angle.

Figure 20 shows the variation of the mean shear rate at different positions along the extrusion direction. Figure 20a indicates that as the thickness of the die increases, the shear rate of the die wall decreases to a certain extent, indicating that increasing the thickness of the die can improve the surface quality of the extruded tread. From Figure 20b, it can be found that the influence of the die convergence angle on the wall shear rate is significant: with the increase of the die convergence angle, the mean shear rate at the wall of the die entrance area decreases significantly, from 132 s^−1^ at α = 3° decreases to 36 s^−1^ at α = 23°, while the mean shear rate at the exit wall of the die increases significantly from 35 s^−1^ at α = 3° to 93 s^−1^ at α = 23°. Because the surface quality of the extruded tread is ultimately determined by the flow state of the rubber in the die outlet area, increasing the die’s convergence angle will greatly reduce the surface quality of the extruded tread.

## 4. Conclusions

In this study, co-extrusion experiments established and verified a viscoelastic numerical simulation model of a tri-composite tread co-extrusion process. In addition, the influence of process parameters and die structure on the co-extrusion process was investigated. The outcome of the study is summarized as follows:

1. The size error of the extruded tread profile between simulation and experiment is less than 6%, which indicates that the numerical simulation technology can be used to predict the profile of the extruded tread, provide theoretical guidance for the structural design of the die thus improve the efficiency of the die design.
2. The difference between the rubber’s rheological properties and the die structure makes the die swelling process and flow characteristics of the three rubbers quite different. Therefore, it is necessary to reasonably design the die structure to prevent the rubber melt’s poor fluidity and excessive flow fluctuation.
3. The die swell rate is directly proportional to the inflow rate and inversely proportional to the traction speed. Increasing the inflow rate will lead to a decrease in the uniformity of rubber flow and the surface quality of the extruded tread, so it is necessary to control the inflow rate within a reasonable range. Die structure has little effect on the extruded tread profile, and increasing the die thickness can moderately improve the flow uniformity of the rubber compound and the surface quality of the extruded tread. However, increasing the convergence angle of the die will cause the shear rate at the die outlet to increase significantly, resulting in a decrease in the surface quality of the extruded tread and, at the same time, bringing a slight negative impact on the uniformity of the rubber flow. Hence, the die needs to be designed as flat as possible.

Overall, the numerical simulation model can be utilized to visualize the flow characteristics in the co-extrusion process and predict the extruded tire shape, which can provide theoretical guidance to improve the design efficiency of the die plate and optimize the design of process parameters and die structure, thus improve the production efficiency and quality of composite tire tread production line.

## Figures and Tables

**Table 1:** PTT constitutive model parameters.

Rubber	η_0_/(Pa.s)	λ/s	ε	ξ
TC	142,708	10.8	0.019	0.19
TB	116,929	9.5	0.014	0.21
TW	202,837	11.6	0.022	0.14

**Table 2:** Process parameters.

Rubber	Inflow Rate (m^3^/s)	v_z_ (m/s)
TC	2.5 × 10^−4^	0.51
TB	5.7 × 10^−5^	0.51
TW	2.1 × 10^−5^	0.51

**Table 3:** The geometric error of simulation tread profile.

	H_1_/mm	W_1_/mm	H_2_/mm	W_2_/mm	W_3_/mm	Die Swell Rate/%
Experimental	6.38	39.1	75.9	8.27	102.4	26.2
Simulation	6.28	37.3	73.8	8.45	102.1	24.9
Deviation rate/%	3.2	5.9	5.5	4.3	1.3	5.8

**Table 4:** Process parameters plan.

Plan	1	2	3	4	5
Inflow rate	0.8 Q	1.2 Q	1.0 Q	1.0 Q	1.0 Q
Traction speed	1.0 v	1.0 v	1.0 v	0.8 v	1.2 v

**Table 5:** Die structures plan.

Plan	b	c	a	d	e
Thickness T/mm	15	35	25	25	25
Converge angle α/°	3	3	3	13	23

## Data Availability

Not applicable.
